# A Word to Students on the Importance of Mechanical Dentistry

**Published:** 1892-03

**Authors:** Guy Harper


ARTICLE V.

### A WORD TO STUDENTS ON THE IMPORTANCE OF MECHANICAL DENTISTRY.

BY GUY HARPER, L. D. S.

Looking back on the examinations of past years one cannot help noticing the greater range of subjects that are nowadays required to satisfy the examiners for the L. D. S., Eng., diploma. Indeed, it is only of late years that the question of mechanical dentistry has found its way into the papers in any serious form, and this is the more to be wondered, as, seeing what splendid mechanics some of the older dentists were; but no, for some reason or other this matter was almost totally ignored, at any rate in the practical part of the examination; lately, however, it has been considered of so much importance that the hospitals have been obliged to provide laboratories where the students may acquire the degree of excellence necessary to pass their examination. Now this brings us to the all-important question of pupildom; one may say that there are three classes of pupils, one, and let us hope the largest, that *will* work, another who think that the only way to acquire a competent knowledge of mechanical dentistry is by manufacturing articles of *vertu* for his lady friends, and lastly the pupil who takes into himself the manner of a chrysalis only to singe his wings when he has developed into the full bloom of a medical student. Three years is none too long to master all the minutiæ of our work, even though constant attention be paid throughout the whole of that period. For the time being let us consider dentistry as a trade; in other trades pupils do not start on their own account directly they are out of their articles, whereas a dentist, as often as not does, for after all the hospital is only another period of apprenticeship, sometimes even we find the two periods run into one, the hospital and the workroom attended at one and the same time. Surely, we, who are supposed to be masters of practically a trade and a profession, have more knowledge and a greater delicacy of touch to acquire than a smith or a carpenter. Ask one of these "of what use is an apprentice at the end of four years," and when you hear his reply think that some dentists are considered competent to practice after four and only four years of study. It is an established fact that many men are apt—too apt to consider the mechanical side of our work as beneath their dignity, and that operative work possesses charms that the workroom does not; still, for all that, dentists and embryo dentists should bear in mind that the term dentistry includes both branches of the work, and that both the operator and mechanic have equal rights to the name. Certainly much of your future success will depend on the careful study of laboratory matters; it trains the hand as nothing else can. A good mechanic will be found, generally speaking, to be a good operator, and the British public are better judges of mechanical than operative work. During the years of their apprenticeship, pupils should remember that it does not fall to everyone's lot to practice in London, and that many are forced by circumstances, over which they have no control, to practice in the country where no work is done for the profession. Then vain regrets for lost time and opportunities take the place of the much wanted gold stoppings that once were considered the sum total of a dental education.

The technical part of an industrial pursuit can be *learned*, principles alone can be *taught*. To learn the trade of husbandry, the agriculturist must serve an apprenticeship to it; to inform his mind in the principles of science he must frequent a school specially devoted to this subject. It is impossible to combine the two, they must be taken up successively, and careful study devoted to each.—*Dental Record.*

